# Senescence Alters Antimicrobial Peptide Expression and Induces Amyloid‐β Production in Retinal Pigment Epithelial Cells

**DOI:** 10.1111/acel.70161

**Published:** 2025-07-13

**Authors:** Jian Liu, Caijiao Yi, Jinyan Qi, Xuexue Cui, Xiangling Yuan, Wen Deng, Mei Chen, Heping Xu

**Affiliations:** ^1^ Aier Academy of Ophthalmology Central South University Changsha Hunan China; ^2^ Aier Eye Institute Changsha Aier Eye Hospital Changsha Hunan China; ^3^ The Wellcome‐Wolfson Institute for Experimental Medicine Queen's University Belfast Belfast UK

**Keywords:** ageing, blood‐retinal barrier, immune regulation, infectious, microbiota, para‐inflammation, retinal degeneration

## Abstract

Age‐related retinal degeneration, such as diabetic retinopathy and age‐related macular degeneration, are major causes of blindness in modern society. Recent studies suggest that dysbiosis and intraocular translocation of bacteria from the blood circulation are critically involved in retinal degeneration. We hypothesise that the blood‐retinal barrier (BRB) cells can protect the neuroretina from blood‐borne pathogens by producing antimicrobial peptides (AMPs). The antimicrobial activity may decline during ageing, putting the retina at risk of low‐degree chronic inflammation and degeneration. Here, we found that the retinal pigment epithelial (RPE) cells, which form the outer BRB, express a variety of AMPs/AMP precursors, including *APP*, *RARRES2*, *FAM3A*, *HAMP*, *CAMP*, *GNLY*, and *PI3*. Senescent RPE cells expressed lower levels of *APP* and *RARRES2* mRNA, accompanied by increased intracellular retention of *E. coli* in a bactericidal assay. Silencing *APP*, not *RARRES2*, with shRNA reduced the antibacterial activity of RPE cells. Senescent RPE cells had lower levels of α‐secretase and higher levels of β‐secretase (*BACE1*) and γ‐secretase (*PS1*), accompanied by reduced soluble APPα and increased amyloid beta (Aβ) production, particularly the Aβ42 isoform. Eyes from aged donors showed a higher Aβ accumulation within RPE cells. Our results suggest that while RPE cells possess antimicrobial activity, this ability declines with age and is impaired in senescent cells. The impaired antimicrobial activity and augmented Aβ deposition in senescent RPE cells may contribute to age‐related retinal para‐inflammation and neurodegeneration.

## Graphical abstract

Healthy RPE cells produce AMPs and AMP precursors, including amyloid precursor protein (APP) and chemerin, and protect the neuroretina from blood‐borne pathogens and maintain homeostasis. When RPE cells undergo senescence, the antibacterial activity declines, and APP undergoes amylogenesis, leading to Aβ deposition. The reduced barrier function of RPE cells, along with Aβ deposition, induces cell damage and para‐inflammation, putting the retina at risk of developing age‐related degenerative diseases.

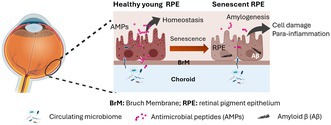

## 1 Introduction

The neuroretina is an immune‐privileged tissue due to the physical (blood‐retinal barrier, BRB) and immunological barriers (Forrester et al. 2008). The BRB is formed by the tight junctions between retinal vascular endothelial cells (inner BRB) and retinal pigment epithelial (RPE) cells (outer BRB: oBRB). The barrier cells, particularly RPE cells and retinal neurons, express and release various immune regulatory or immune suppressive molecules, constituting the immunological barrier. This extremely sophisticated defence system minimises inflammation‐mediated retinal damage and disease. Despite the high levels of immune regulation, retinal inflammation (e.g., infectious and non‐infectious) and inflammation‐related retinal degeneration remain a major cause of blindness, and the underlying mechanisms remain poorly defined.

Age‐related Macular Degeneration (AMD), Diabetic Retinopathy (DR), and glaucoma are typical examples of inflammation‐related retinal degeneration. AMD affects individuals over the age of 55 and is the major cause of blindness in the elderly in developed countries (Mitchell et al. 2018). DR affects up to 80% of all patients who have had diabetes for 10 years or more (Fong et al. 2004). According to the World Health Organisation, over 20 million people are visually impaired globally due to DR, glaucoma, or AMD (Burton et al. 2021). Although the initial cause of the disease differs in DR, AMD, and glaucoma, dysregulated and uncontrolled intraocular inflammation is known to play a critical role in all conditions.

Genetic predisposition, ageing, and environmental factors (e.g., lifestyle, diet) are major risk factors for retinal degenerative diseases such as AMD, DR, and glaucomatous retinopathy. We previously showed that immune regulation in the ageing retina is dysregulated, leading to a low‐grade chronic inflammation (para‐inflammation) (Chen et al. 2019; Xu et al. 2009). The dysregulated para‐inflammatory response puts the ageing retina at risk of developing degenerative diseases (Chen et al. 2019). How the risk factors work together, leading to dysregulated intraocular inflammation and retinal degeneration, remains unknown. Recent advances in the role of microbiota in human health and disease, and the discovery of the gut‐eye axis, suggest that environmental factors may affect retinal health by altering the microbiome and the immune system (Wen et al. 2018). Emerging evidence suggests that many retinal degenerative conditions have an infectious origin. For example, *Chlamydia pneumoniae* infection is related to the risk of AMD (Robman et al. 2005). *Helicobacter pylori* infection increases the risk of DR (Liu et al. 2023) and glaucoma (Ezzati Amini and Moradi 2023). Virus infection (e.g., hepatitis B/C, cytomegalovirus, COVID‐19) is a risk factor for AMD, DR, and glaucoma (Lyons et al. 2023; Wu et al. 2019; Yeh et al. 2021).

Recently, bacteria‐specific DNA and culturable bacteria have been found in healthy human blood (Panaiotov et al. 2021). Circulating bacteroides abundance was higher in healthy people than in those with cardio‐metabolic disorders (Ullah Goraya et al. 2023) and may play a role in the pathogenesis of non‐infectious diseases such as diabetes, cardiovascular diseases, and neurodegenerative diseases (Ullah Goraya et al. 2023). Furthermore, a disease‐specific intraocular microbial signature was detected in people with AMD and glaucoma (Deng et al. 2021), suggesting that spontaneous or pathogenic bacterial translocation from blood or other sources may be associated with these common sight‐threatening conditions. Indeed, a recent study has shown that photoreceptor degeneration in the Crb1 mutant mice is dependent on intraocular bacterial translocation (Peng et al. 2024).

We previously reported that RPE cells constitutively express a high level of antimicrobial peptide (AMP), lysozyme (Liu et al. 2021), suggesting that the oBRB is also a barrier that prevents the invasion of blood‐borne pathogens. In this study, we further examined the AMP expression profile of RPE cells and investigated the impact of cell senescence on the expression and production of AMPs/AMP precursors in RPE cells.

## 2 Methods

### 2.1 Single‐cell RNA sequencing (scRNA‐seq) and Single‐Nucleus RNA‐sequencing (snRNA‐seq ) analysis of the expression profile of AMPs / AMP precursors in RPE cells

For the single‐cell RNA sequencing (scRNA‐seq) and single‐nucleus RNA sequencing (snRNA‐seq) analysis of the expression profile of AMPs/AMP precursors in RPE cells, we used the single‐cell RNA sequencing dataset (GSE210543) (Collin et al. 2023) from human RPE/choroid samples and the online platform, Spectacle (https://singlecell‐eye.org/app/spectacle/) (Voigt et al. 2020) to analyse the AMP profile in ocular cells. Thirteen scRNA‐seq datasets were merged into one gene × cell counts matrix. The visualisation of AMP expression in snRNA‐seq of RPE cells was retrieved from the Single Cell Portal (https://singlecell.broadinstitute.org/) and single‐nucleus RNA sequencing dataset (GSE135133), from the eyeballs of five donors using snRNA‐SEQ with 10× Genomics Chromium Single Cell 3′ Gene Expression Kit (Orozco et al. 2020).

### 2.2 Cell Culture and Treatment

The human RPE cell line ARPE19 cells were cultured in DMEM/F12 medium supplemented with 10% fetal bovine serum and 1% penicillin‐streptomycin (Thermo Fisher Scientific, Shanghai, China). Primary mouse RPE cells were isolated from mouse eyes and cultured in the DMEM medium containing 10% FBS and 1% penicillin‐streptomycin using the protocol described in our previous publication (Chen et al. 2008). Cells from the first 5 passages were used in the study.

The ARPE19 cells were treated with lipopolysaccharide (LPS, 100 ng/mL; Sigma‐Aldrich, Shanghai, China), Pam3CSK4 (2 ng/mL, Sigma‐Aldrich), *staphylococcus aureus* ‐derived peptidoglycan (PGN‐SA, 10 μg/mL, InvivoGen), Poly(I:C) (5 μg/mL, InvivoGen, San Diego, CA, USA) alone or transfected with Lipofectamine 2000 (Lipo2000, Thermo Fisher Scientific), and Poly (dA:dT) (5 μg/mL, InvivoGen) alone or transfected for 24 h according to our previous study (Liu et al. 2021). The cells were then processed to evaluate AMP expression (see below).

**Induction of cell senescence**: ARPE19 cells (4 × 10^5^) were seeded into a 6‐well plate and were exposed to doxorubicin (Dox, 0.5 μg/mL, Sigma‐Aldrich) or hydrogen peroxide (H_2_O_2_, 100 μM, Sigma‐Aldrich). The Dox was removed, cells were washed, and fresh media were added 24 h later. The H_2_O_2_ was refreshed daily for 5 days. Total RNA or proteins were extracted for further investigations.

### 2.3 Knockdown of *APP* and *RARRES2*

To silence the expression of the *APP* and *RARRES2* genes in ARPE19 cells, shRNA oligonucleotides targeting human *APP* (Forward: ACCGGTGCCATCTTTGACCGAAACGAACT CGAGTTCGTTTCGGTCAAAGATGGCTTTTTGGAATTC) and *RARRES2* (Forward: ACCGGTGCTGGAATTTAAGCTGCAGCACTCGAGTGCTGCAGCTTAAATTCCAGCTTTTTGGAATTC) were annealed in a T4 ligase buffer and directionally cloned into AgeI/EcoRI‐digested pLKO.1 vectors. The recombinant plasmids were validated through colony PCR and Sanger sequencing and purified via an endotoxin‐free plasmid extraction kit (Omega, Norcross, GA, USA). Subsequently, purified plasmids were transfected into ARPE19 cells using Lipofectamine 2000 in the Opti‐MEM medium. The knockdown efficiency was validated by real‐time qPCR. The cells were then used in bactericidal assays.

### 2.4 β‐Galactosidase (β‐Gal) Staining

β‐galactosidase staining was conducted using the Senescence β‐Galactosidase Staining Kit (Beyotime, Shanghai, China) according to the manufacturer's instructions. Briefly, the cells were washed and fixed with the fixative solution at room temperature for 15 min, followed by incubation with the β‐gal staining solution overnight at 37°C.

### 2.5 Real‐Time Quantitative PCR (qPCR)

Total mRNA was extracted using the Promega RNA extraction kit (Promega, Madison, WI). The same amount of RNA was reverse transcribed into cDNA as a template using the reverse transcription kit. The PCR reaction mixture included 10 ng of the cDNA and ChamQ Universal SYBR qPCR Master Mix (Vazyme Biotech, Nanjing, China). The primers used in the study are listed in Table 1.

**TABLE 1:** Primer sequences of human and mouse AMP and beta‐actin genes.

Gene	Sequence	Gene bank access no.
Human Actb	Forward: 5′‐ACAGAGCCTCGCCTTTGC‐3′	NM_001101.5
	Reverse: 5′‐ATCACGCCCTGGTGCCT‐3′	
Human GNLY	Forward: 5′‐TGCTCCTGGGCAACCC‐3′	NM_001302758.2
	Reverse: 5′‐GGGTGGGCTTATCCACCATC‐3′	
Human Hamp	Forward: 5′‐TTTTCCCACAACAGACGGGA‐3′	NM_021175.4
	Reverse: 5′‐CTCCTTCGCCTCTGGAACAT‐3′	
Human PI3	Forward: 5′‐TGTTGAATCCCCCTAACCGC‐3′	NM_002638.4
	Reverse: 5′‐GAAGGGCAGCAGGGACTTAG‐3′	
Human CAMP	Forward: 5′‐TGACTTCAAGAAGGACGGGC‐3′	NM_004345.5
	Reverse: 5′‐AGGGCACACACTAGGACTCT‐3′	
Human APP	Forward: 5′‐TGGAGGTACCCACTGATGGT‐3′	NM_000484.4
	Reverse: 5′‐GCACCAGTTCTGGATGGTCA‐3′	
Human RARRES2	Forward: 5′‐GGAGGAAACGGAAATGCCTG‐3′	NM_002889.4
	Reverse: 5′‐AAGGCGAACTGTCCAGGGAA‐3′	
Human P53	Forward: 5′‐GGACAGCCACGTCTGTGACTTG‐3′	PP845877.1
	Reverse: 5′‐CCAGTGGTTTCTTCTTTGGCTG‐3′	
Human P21	Forward: 5′‐CCTGCCCAAGCTCTACCTT‐3′	NM_078467.3
	Reverse: 5′‐AAGGCAGAAGATGTAGAGC‐3′	
Human Bace1	Forward: 5′‐GCAGGGCTACTACGTGGAGA‐3′	NM_138972
	Reverse: 5′‐GTATCCACCAGGATGTTGAGC‐3′	
Human Ps1	Forward: 5′‐GTGGTTGTTCGTGATCCTTGC‐3′	NM_172341.4
	Reverse: 5′‐TGGCTCTGTTCTGTGTAGGC‐3′	
Human AMAM9	Forward: 5′‐TGTGTCTCCTAGTAGCTTCCT‐3′	NM_003816.3
	Reverse: 5′‐CCATTGTGCACACTTTGGCA‐3′	
Mouse PI3	Forward: 5′‐GAGGACAGTTCTGCTGGGTC‐3′	NM_001395120.1
	Reverse: 5′‐AAGCAGAATGGTCACTGCGA‐3′	
Mouse HAMP	Forward: 5′‐AGGGCAGACATTGCGATACC‐3′	NM_032541.2
	Reverse: 5′‐GCAACAGATACCACACTGGGA‐3′	
Mouse CAMP	Forward: 5′‐CGGCCGCTGATTCTTTTGAC‐3′	NM_009921.2
	Reverse: 5′‐CCACCTTTGCGGAGAAGTCC‐3′	
Mouse APP	Forward: 5′‐CTACGGAAACGACGCTCTCA‐3′	NM_001198823.1
	Reverse: 5′‐CAGAACCTGGTCGAGTGGTC‐3′	
Mouse RARRES2	Forward: 5′‐TACAGGTGGCTCTGGAGGAGTTC‐3′	NM_027852
	Reverse: 5′‐CTTCTCCCGTTTGGTTTGATTG‐3′	
Mouse FAM3A	Forward: 5′‐TCATGAGCAGCGTCAAAGAC‐3′	NM_025473.5
	Reverse: 5′‐AGGGTACCTTCATGCAGTGG‐3′	
Mouse Actb	Forward: 5′‐CCTTCCTTCTTGGGTATG‐3′	XM_030254057.1
	Reverse: 5′‐TGTAAAACGCAGCTCAGTAA‐3′	

### 2.6 Human Eye Tissues

The human eyes used in this study were obtained from the Aier Eye Banks (Wuhan Aier Eye Bank and Changsha Aier Eye Bank). The study complies with the Declaration of Helsinki, and the protocol was approved by the Ethical Review Board of Changsha Aier Eye Hospital (Ref: AIER2019IRB12). Eyeballs from donors aged between 42 and 89 years old without known ocular diseases were used in the study. Eyes were collected within 8 h after death and were immediately fixed in Davis fixation solution for 24 h. The tissues were dehydrated using ethanol, embedded in paraffin, and sectioned for immunohistochemical staining.

### 2.7 Immunohistochemistry

The eye sections were deparaffinised with xylene and rehydrated through a graded ethanol series. The samples were treated with Tris‐EDTA buffer at a high temperature for antigen retrieval, followed by incubation with an alkaline phosphatase inhibitor for 15 min, and then in 2% BSA with 5% goat serum for 1 h. Subsequently, the samples were incubated with primary antibodies at 4°C overnight. The antibodies used in the study are detailed in Table 2. The sections were washed and incubated with biotinylated secondary antibodies (goat anti‐mouse or goat anti‐rabbit) at room temperature for 1 h. After thorough washes, alkaline phosphatase–labelled streptavidin was added and incubated at room temperature for 30 min. Colour was developed using the FAST‐Red alkaline phosphatase (AP) substrate (ZSGB Biotechnology) counterstained with Safranin solution. Two independent researchers conducted the quantification of immunohistochemical staining with no knowledge of study groups using colour deconvolution in Fiji software described previously (Crowe and Yue 2019).

**TABLE 2:** Details of sources and concentrations of antibodies used for immunochemistry in this study.

Antibody	Host species	Dilution	Distributor, Cat. no.
Amyloid Precursor Protein antibody	Rabbit	1:500	Abcam, ab32136
Anti‐beta Amyloid 1–42 antibody	Mouse	1:500	Biolegend, 805,509
Cathelicidin/CLP antibody	Rabbit	1:200	Abcam, ab180760
Chemerin Polyclonal Antibody	Rabbit	1:500	ThermoFisher, PA5‐77080
FAM3A Monoclonal Antibody (7‐B9)	Mouse	1:200	ThermoFisher, MA5‐32880
GNLY/Granulysin antibody	Rabbit	1:200	Abcam, ab241333
HAMP Polyclonal Antibody	Rabbit	1:200	ThermoFisher, PA5‐102436
PI3 Polyclonal Antibody	Rabbit	1:200	ThermoFisher, PA5‐35877

### 2.8 Bacterial Killing Assays

The *E. coli* DH5a strain was cultured in a Luria‐Bertani (LB) liquid medium until reaching the logarithmic growth phase (OD_600_ = 0.4–0.6) and then diluted in ddH_2_O. The *E. coli* were added to the ARPE19 cells at a ratio of 1:1 (MOI). The supernatant of ARPE19 cells was collected 6 h later, and the cells were washed five times with sterile PBS to remove the residual *E. coli* . The genomic DNA of ARPE19 cells was subsequently extracted using a DNA extraction kit. Two primers targeting *E. coli* 16s rRNA primers Baclf: Forward‐AAATTGAAGAGTTGATC; Reverse‐TAAGGAGGTGATCCA and Ecol: Forward‐GACCTCGGTTTAGTTCACAGA; Reverse‐ CACACGCTGACGCTGACCA (Miyazaki et al. 2017) were used for RT‐PCR detection of ingested residual *E. coli* . The PCR products were analysed by agarose gel electrophoresis, and the grayscale intensity of bands was quantified using Image J software. The ratio of the band intensity of ingested *E. coli* to total DNA was calculated to determine the bactericidal capacity of ARPE19 cells. On the other hand, the supernatant was diluted in LB medium according to the total cellular protein content, and an appropriate volume was spread onto LB agar plates for overnight incubation, followed by bacterial counting.

### 2.9 Enzyme‐Linked Immunosorbent Assay (ELISA)

The levels of soluble APPα, Aβ1‐40, and Aβ1‐42 in ARPE19 cell lysates or the supernatants were measured by ELISA following the manufacturer's protocol (Cat: CSB‐EQ027464HU, CSB‐E08299H, CSB‐E10684H, CUSABIO Technology, Houston, TX). Briefly, ARPE19 cells were lysed using the RIPA lysis buffer containing protease and phosphatase inhibitors. The protein concentration was determined using the BCA assay. Equal amounts of total protein (cell lysate) and equal volumes of supernatants were separately added into 96‐well plates pre‐coated with anti‐Aβ42, anti‐Aβ40 and anti‐sAPPα antibodies and incubated overnight at 4°C. Unbound proteins were carefully removed by thorough washes, and HRP‐conjugated secondary antibodies were added and incubated at room temperature for 1 h, followed by 100 μL of substrate incubation. The absorbance at 450 nm was measured using a microplate reader.

### 2.10 Statistical Analysis

All experiments were repeated at least three times. The data were processed and analysed using Prism software (version 8.0.2, GraphPad Software). Normality and homogeneity of variance were assessed using the Shapiro–Wilk test (*n* < 50) or Kolmogorov–Smirnov test (*n* ≥ 50) for normality, and the corrected Bartlett's test for variance equality, respectively, with data expressed as mean ± SD (normal distribution) or median ± IQR (non‐normal distribution). One‐way ANOVA was used to compare the differences between three or more groups, followed by Tukey's multiple comparisons post hoc analysis. Two‐way ANOVA was used to compare the differences according to the levels of two categorical variables. The difference between the two groups was analysed using an unpaired *t*‐test.

## 3 Results

### 3.1 Retinal Pigment Epithelial Cells Constitutively Express A Variety of AMPs/AMP Precursors

**FIGURE 1:**
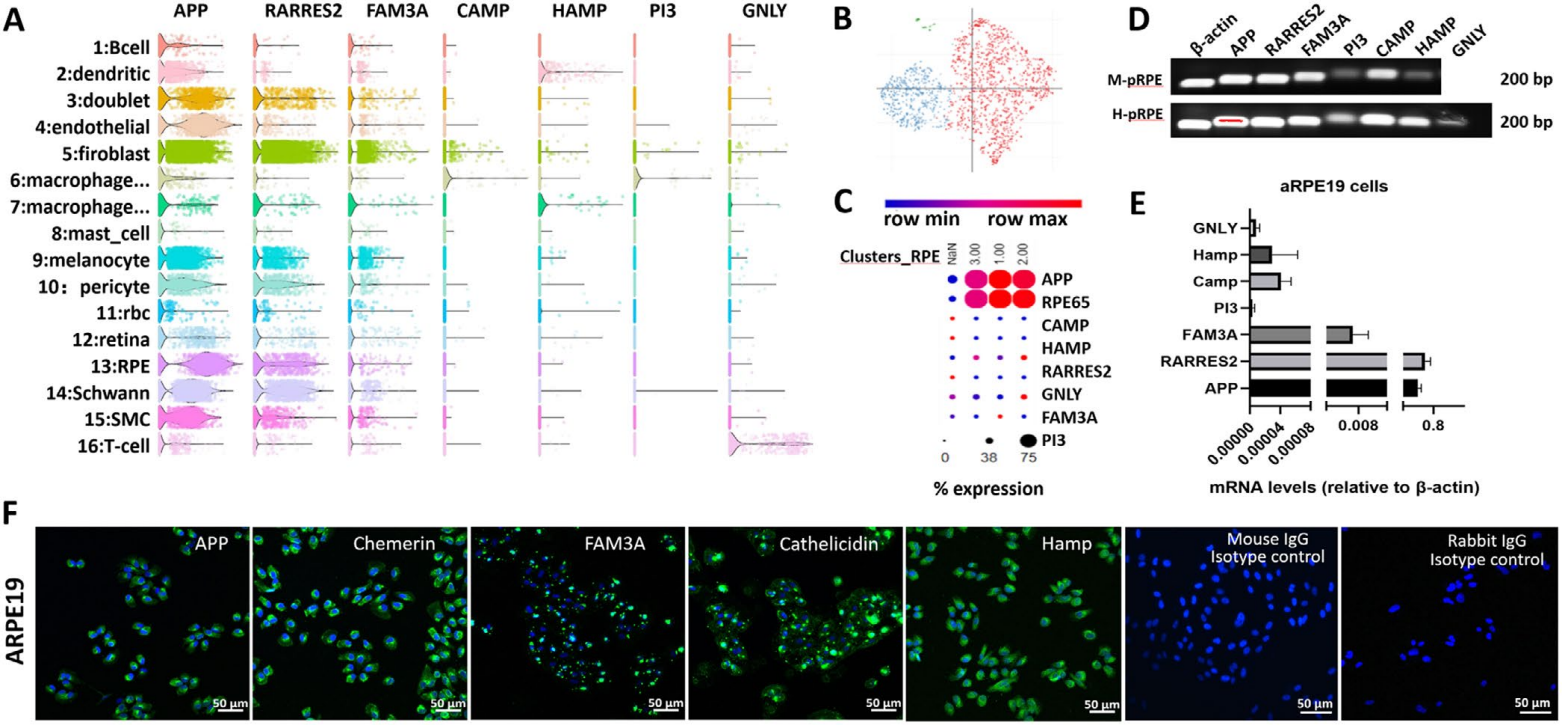
AMP and AMP precursor gene and protein expression in RPE cells. (A) A violin plot showing the expression of AMP genes (*APP, RARRES2, FAM3A, CAMP, HAMP, PI3, GNLY*) in different types of cells from the Single‐cell RNA sequencing dataset of RPE‐Choroidal tissue (GSE210543) using the online platform, Spectacle (Voigt et al. 2020). (B, C) Single‐nucleus RNA sequencing showing the expression of AMPs/AMP precursor genes in different clusters of RPE cells from the dataset (GSE135133). (B) t‐SNE plot of re‐clustered RPE cells. (C) The expression profile (percentage of cells (dot size) and average expression levels (colour intensity)) of various AMPs/AMP precursor genes in different subsets of RPE cells. (D) Semiquantitative RT‐PCR validation of AMPs/AMP precursor gene expression in mouse (M‐pRPE) and human (H‐pRPE) primary RPE cells. (E) Quantitative RT‐PCR analysis of AMPs/AMP precursor mRNA levels in ARPE19 cells, normalised to *Actb*, *n* = 3, Mean ± SD. (F) Immunofluorescence images showing AMPs/AMP precursor protein expression (Green) in ARPE19 cells. Nuclei are stained with DAPI (blue). Scale bars: 50 μm.

Dataset GSE210543 contains 16 clusters of cells, and cluster 13 was classified as RPE cells. *APP* and *RARRES2* were highly expressed in RPE cells, followed by *FAM3A*. Other AMPs, including *CAMP, HAMP, GNLY*, and *PI3*, were also detected in RPE cells (Figure 1A). Dataset GSE135133 has three clusters of RPE cells (Figure 1B). The *APP* was highly expressed in all clusters, and *RARRES2* was moderately expressed in clusters 2 and 3, whereas *FAM3A* and *PI3* were detected in clusters 2 and 1, respectively (Figure 1C).

To further confirm the scRNA‐seq results, we conducted RT‐PCR and qPCR in primary human and mouse RPE cells as well as in the ARPE19 cells. RT‐PCR showed that *APP, RARRES2, FAM3A*, and *CAMP* were highly expressed in human and mouse RPE cells (Figure 1D). Human primary RPE cells also expressed high levels of *HAMP* but low levels of *PI3* and *GNLY* (Figure 1D). qPCR showed ARPE19 cells expressed high levels of APP and RARRES2, followed by *FAM3A, CAMP, HAMP, PI3*, and *GNLY* (Figure 1E). The expressions of *APP, RARRES2* (Chemerin), *FAM3A, CAMP* (Cathelicidin), and *HAMP* were further confirmed at the protein level by immunocytochemistry in ARPE19 cells (Figure 1F). Cells incubated with isotype control antibodies did not show any immunoreactivity (Figure 1F). APP and chemerin (encoded by *RARRES2*) are AMP precursors as they have no direct bactericidal effect, but their proteolytic fragments (small peptides) have antimicrobial functions (Soscia et al. 2010; Sozzani et al. 2024). Our results suggest that RPE cells constitutively express a variety of AMPs/AMP precursors.

### 3.2 AMP/AMP Precursor Gene Expression in RPE Cells in Inflammatory Conditions

To understand how the expression of AMPs/AMP precursors is regulated under inflammatory or infectious conditions, we treated ARPE19 cells with various TLR/NLR agonists, including synthetic double‐stranded DNA poly(dT:dT), synthetic double‐stranded RNA (poly(I:C)) (TLR3 agonist), PAM3CSK (TLR1/2 agonist), LPS, and PNG‐SA (Figure S1).

The poly(dA:dT) treatment significantly reduced the expression of F*AM3A, CAMP*, and *HMAP*. Interestingly, poly(I:C) enhanced the expression of *APP* and *GNLY* but reduced the expression of *FAM3A*. When poly(I:C) was transfected into RPE cells with Lipo2000, it significantly increased the expression of *CAMP* and *HAMP* and decreased the expression of *RARRES2*. LPS reduced the expression of *CAMP, HAMP*, and *GNLY*. Pam3CSK reduced the expression of *FAM3A* and *GNLY* and increased the expression of *RARRES2*. PGN‐SA promoted the expression of *APP* (Figure S1). Our results suggest that different pathogens may induce the expression of different AMPs at the oBRB.

### 3.3 AMP/AMP Precursor Expression in Senescent RPE

**FIGURE 2:**
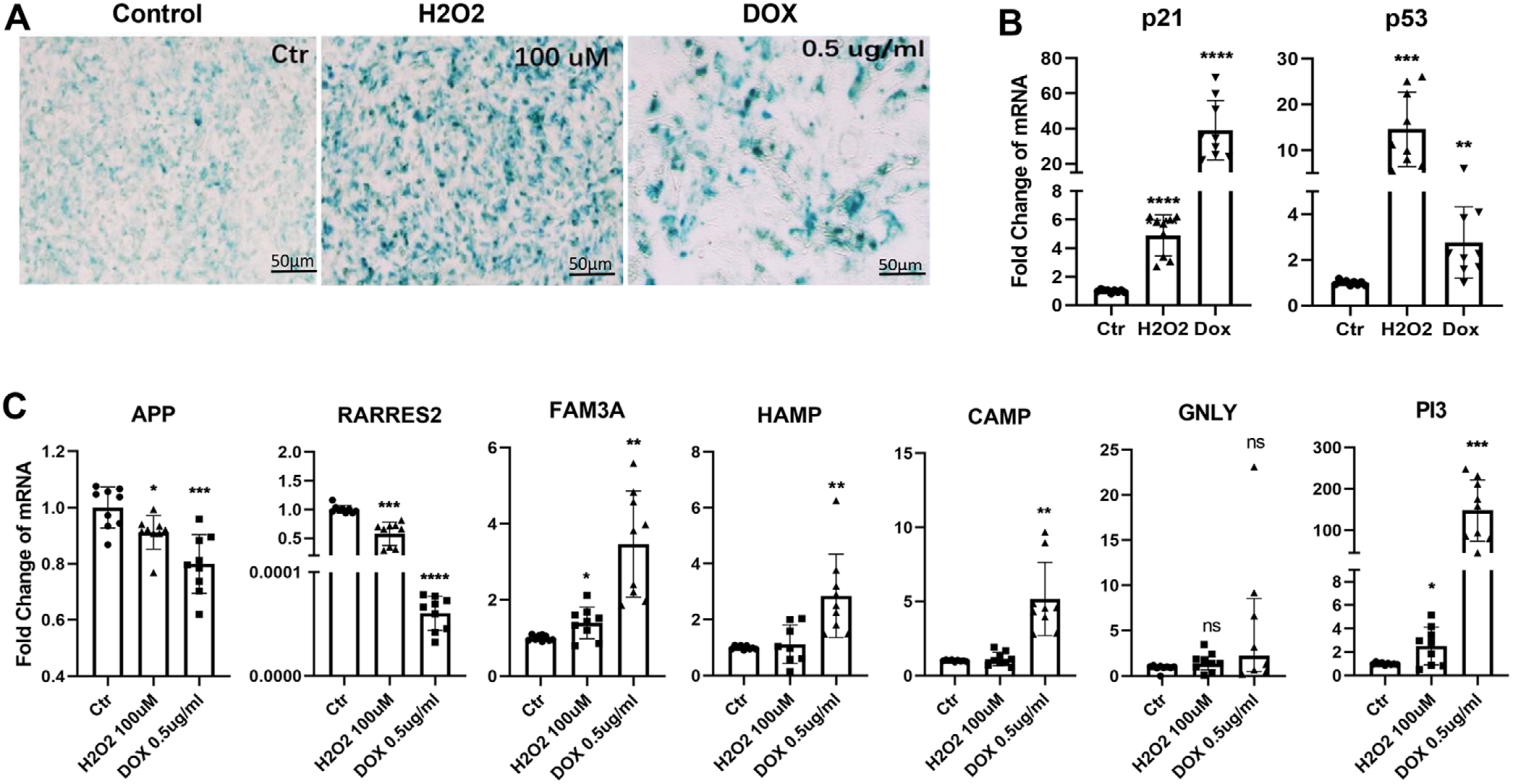
The AMP mRNA expression in senescent ARPE19 cells. ARPE19 cells were treated with H_2_O_2_ (100 μM) or doxorubicin (Dox, 0.5 μg/mL) for 5 days. (A) Representative images of senescence‐associated β‐galactosidase (SA‐β‐gal) staining in different groups of cells. Scale bars = 50 μm. (B) Quantification of mRNA expression of senescence markers *p21* and *p53* in control (Ctr), H_2_O_2_‐treated, and Dox‐treated cells. *P21*: Welch's *W* (2.000, 10.67) = 52.30, *p* < 0.0001. Post hoc: Ctr vs. H_2_O_2_, *p* < 0.0001; Ctr vs. DOX, *p* < 0.0001. *p53*: Welch's *W* (2.000, 10.72) = 17.15, *p* = 0.0005. Post hoc: Ctr vs. H_2_O_2_, *p* = 0.0001; Ctr vs. DOX, *p* = 0.0039. (C) Real‐time qPCR to detect mRNA expression levels of AMP precursor/AMP genes in control, H_2_O_2_‐treated, and Dox‐treated cells. *APP*: Welch's *W* (2.000, 15.34) = 11.03, *p* = 0.0011. Post hoc: Ctr versus H_2_O_2_, *p* = 0.0237; Ctr versus DOX, *p* = 0.0006. *RARRES2*: Welch's *W* (2.000, 9.805) = 148.7, *p* < 0.0001. Post hoc: Ctr versus H_2_O_2_, *p* = 0.0003; Ctr versus DOX, *p* < 0.0001. *FAM3A*: Welch's *W* (2, 11.02) = 6.85, *p* = 0.004. Post hoc: Ctr versus H_2_O_2_, *p* = 0.0385; Ctr versus DOX, *p* = 0.0013. Hamp: Welch's *W* (2.000, 10.06) = 6.576, *p* = 0.0149. Post hoc: Ctr versus DOX, *p* = 0.0107. CAMP: Welch's *W* (2.000, 10.82) = 2.35, *p* = 0.0016. Post hoc: Ctr versus DOX, *p* = 0.0018. PI3: Welch's *W* (2.000, 10.70) = 20.24, *p* = 0.0002. Post hoc: Ctr versus H_2_O_2_, *p* = 0.0403; Ctr versus DOX, *p* = 0.0007. Data are shown as fold change relative to controls in B and C; *n* = 9, Mean ± SD. *p < 0.05, **p < 0.01, ***p < 0.001, ****p < 0.0001. The normality of data distribution and variance homogeneity were assessed by Shapiro–Wilk test and Bartlett's test, respectively, and Welch's ANOVA test with Dunnett's T3 multiple comparisons test.

To investigate the effects of cell senescence on AMP/AMP precursor expression, ARPE19 cells were treated with H_2_O_2_ (100 μM) or Dox (0.5 μg/mL) for 5 days. The senescent phenotype was confirmed by senescence‐associated β‐galactosidase (SA‐β‐gal) staining (Figure 2A) and *p53* and *p21* gene expression (qPCR) (Figure 2B). SA‐β‐gal catalyses the hydrolysis of the substrate X‐Gal (5‐bromo‐4‐chloro‐3‐indolyl‐β‐D‐galactopyranoside), generating an insoluble blue indigo precipitate. ARPE19 cells treated with H_2_O_2_ and doxorubicin (Dox) exhibited more intense blue staining (Figure 2A). Moreover, the expression of p*21* and *p53* increased by 4.8‐ and 14.5‐fold in H_2_O_2_‐treated cells and 28.1‐ and 2.7‐fold in Dox‐treated cells, respectively (Figure 2B).

The expression of *APP* and *RARRES2* was significantly decreased in senescent RPE cells, whereas *FAM3A, HAMP, CAMP*, and *PI3* genes demonstrated different degrees of increase (Figure 2C). The expression of *GNLY* was not significantly affected by cell senescence (Figure 2C).

**FIGURE 3:**
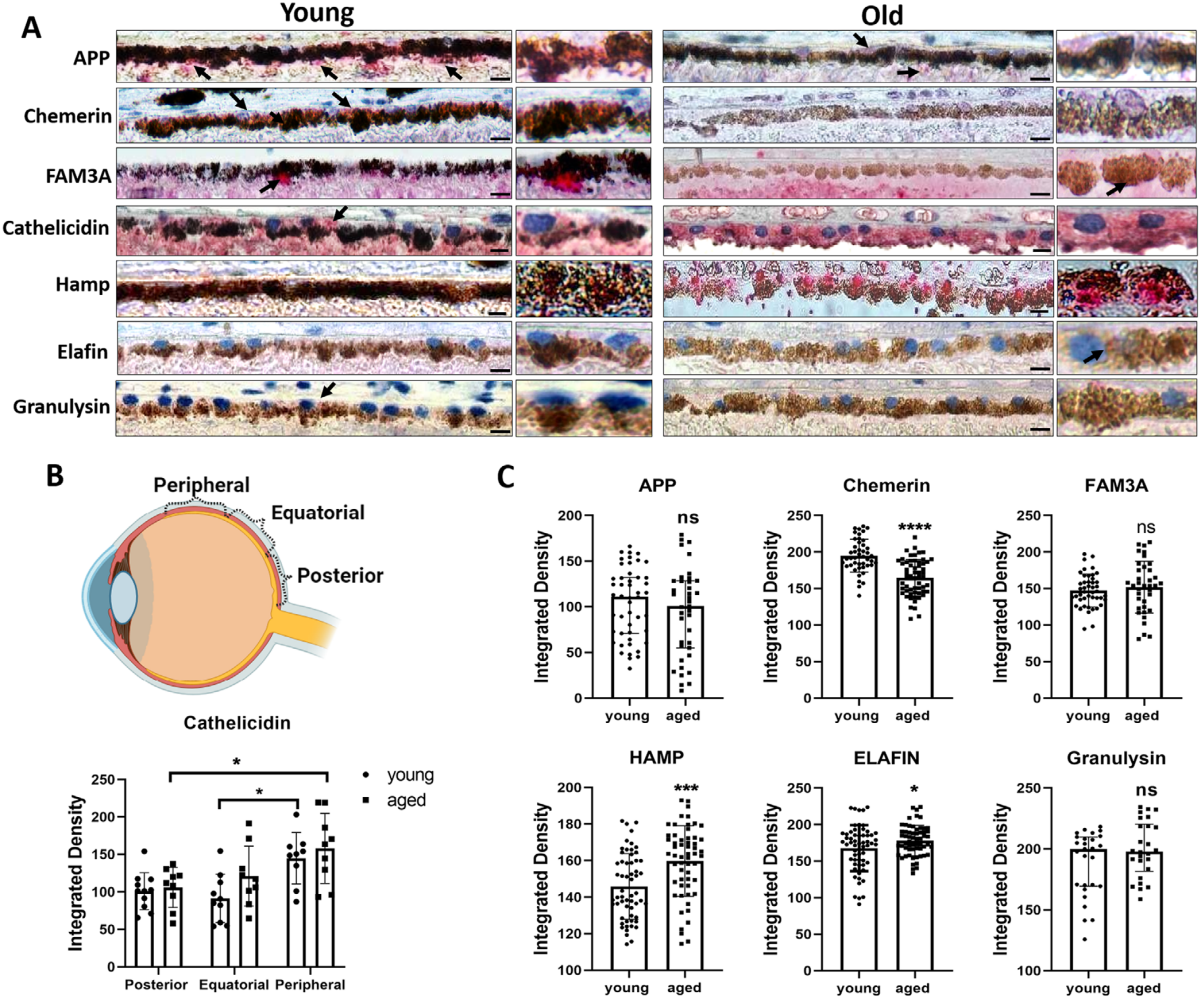
Immunohistochemical staining of AMPs/AMP precursors in young and old human retinal tissues. (A) The panels show the expression and localisation of AMP precursors APP and Chemerin (*RARRES2*), and AMPs including FAM3A, Cathelicidin (*CAMP*), Elafin (*PI3*), Hamp and Granulysin (*GNLY*) in RPE cells from young (left column) and old (right column) donors. FAST Red staining (red) with haematoxylin counterstain (blue). The arrows indicate positive colour development. Scale bars = 20 μm. Ch: Choroid; RPE: Retinal pigment epithelia. (B) Quantification of Cathelicidin expressions in different regions of RPE cells of human eyes, *n* = 12–15 images from 4 eyes, Mean ± SD, Data were analysed by two‐way ANOVA, effect of region (row factor: *F* (2,51) = 11.24, *p* < 0.0001), interaction (*F* (2,51) = 0.64, *p* = 0.5295), age effect (column factor: *F* (1,51) = 3.00, *p* = 0.0896). Tukey's post hoc tests: Equatorial young versus Peripheral young: Mean difference = −53.54, *p* = 0.0169; Posterior aged versus Peripheral aged: Mean difference = −52.06, *p* = 0.0272. (C) Quantitative analysis of immunohistochemical staining (FastRed) by Fiji software. The normality of data distribution was assessed by Normality and Log Normality Tests. Data normally distributed (Chemerin, FAM3A, Hamp, Elafin) are expressed as mean ± SD and analysed by unpaired *t*‐test. *p < 0.05, ***p < 0.001, ****p < 0.0001, ns: No significant difference. Chemerin: *F* (57,44) = 1.22, *p* = 0.491, unpaired *t*‐test, *t* (101) = 6.30, *p* < 0.0001. FAM3A: *F* (36,44) = 2.452, *p* = 0.0049, Welch's corrected *t*‐test: *T* (58.78) = 0.68, *p* = 0.497. Hamp: *F* (56, 57) = 1.150, *p* = 0.5995, unpaired *t*‐test, *t* (113) = 4.002, *p* = 0.0001. Elafin: *F* (57, 59) = 2.312, *p* = 0.0017. Welch's corrected *t*‐test, *t* (98.07) = 2.157, *p* = 0.0335. Granulysin: *F* (26, 25) = 1.341, *p* = 0.4658, unpaired *t*‐test, *t* (51) = 1.723, *p* = 0.0909. Non‐normally distributed data (APP and Granulysin) are presented as median ± interquartile range (IQR) and analysed by the Mann–Whitney *U* test. APP: Young (median = 111.0, *n* = 48) vs. Aged (median = 100.5, *n* = 39), *U* = 823, *p* = 0.3387, Hodges‐Lehmann estimate of median difference (HL Δ) = −10.78; GNLY: Young (median = 199.9, *n* = 27) versus Aged (median = 1197.9, *n* = 26), *U* = 283, *p* = 0.2319, HL Δ = 10.63). *p < 0.05, ***p < 0.001, ****p < 0.0001, ns: No significant difference.

Immunohistochemistry confirmed the expression of these AMPs/AMP precursors in healthy donor eyes (Figure 3A). Positive punctate staining of APP, chemerin, FAM3A, and cathelicidin (arrows) was detected in RPE cells. Hamp, Elafin, and granulysin showed weak staining in RPE cells (Figure 3A, left panel). Cathelicidin had a regional‐specific distribution, that is RPE cells in the peripheral area expressed higher levels of cathelicidin than those in the posterior and equator (Figure 3B). Other AMPs/AMP precursors had equal distribution in different regions. We further compared the expression levels of AMPs/AMP precursors between young (20–50 years old) and old (60–90 years old) donor eyes (Figure 3B,C). RPE cells in the ageing eye were less pigmented and had occasional discontinuity (Figure 3A, right panel). The expression level of chemerin was significantly reduced, and the expressions of Hamp and Elafin were increased in RPE cells from old donors compared to those from young donors (Figure 3C). The results are in line with the in vitro observation in senescent RPE cells (Figure 2).

Taken together, our results suggest that the expression of these AMPs/AMP precursors is affected in senescent RPE cells. The constitutively highly expressed AMP precursor chemerin was down‐regulated, whereas the weakly expressed AMPs such as Hamp and Elafin were upregulated under senescence conditions.

### 3.4 AMP/AMP Precursor Expression and Bactericidal Activity of RPE

**FIGURE 4:**
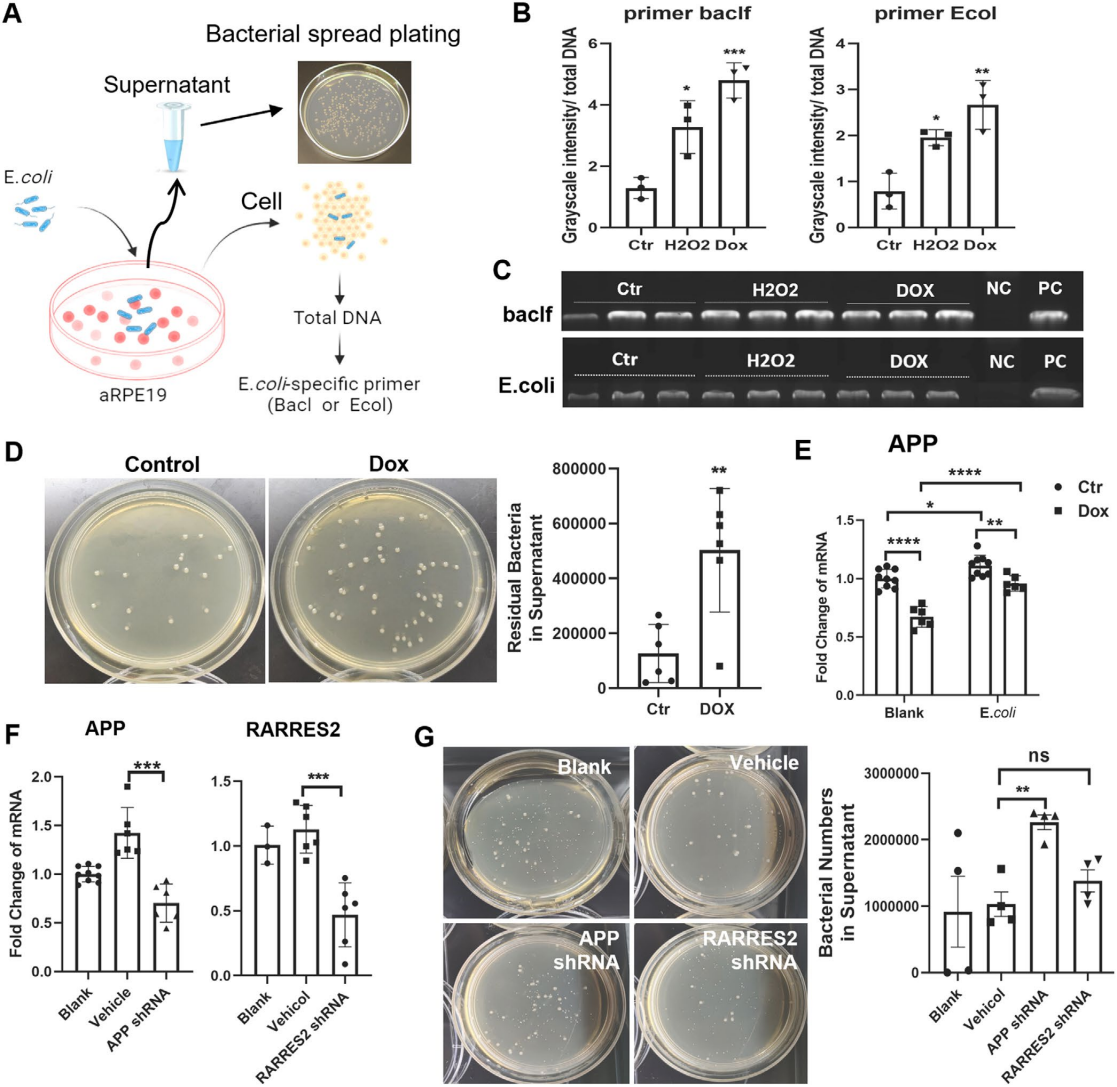
The impact of senescence on bactericidal activity in ARPE19 cells. (A) Schematic graph showing experimental setup. *E. coli* was incubated with ARPE19 cells for 6 h, the supernatant was removed by thorough washes, followed by total DNA extraction and PCR amplification using *E. coli* ‐specific primers (BacI or EcoI). (B) Quantification of PCR product intensity normalised to total DNA for EcoI and BacIf primers, *n* = 3, Mean ± SD, BacIf: *F* (2,6) = 23.50, *p* = 0.002, *R* ^2^ = 0.89. Ctr versus H_2_O_2_, *p* = 0.015; Ctr versus Dox, *p* < 0.001. *E. coli*: *F* (2, 6) = 17.47, *p* = 0.003, *R* ^2^ = 0.8534; Ctr versus H_2_O_2_, *p* = 0.020; Ctr versus Dox, *p* = 0.002. *p < 0.05, **p < 0.01, ***p < 0.001. One‐way ANOVA with Tukey's multiple comparisons post hoc analysis. Ctr: Control; H_2_O_2_: H_2_O_2_‐induced senescent RPE cells; DOX: Doxorubicin‐induced senescent RPE cells. (C) Gel electrophoresis images showing PCR products amplified with BacIf and EcoI primers from different groups. NC: Negative control, that is DNA from ARPE19 cells without bacterial treatment, served as a template. PC: Positive control, that is DNA from bacteria *E. coli* served as a template. (D): Representative images (left) and quantitative analysis (right) of extracellular bacterial colonies in control (Ctr) and Dox‐treated RPE cultures, *n* = 6, Mean ± SD. Welch's paired *t* test, *t* (7.13) = 3.71, *p* = 0.007, *η* ^2^ = 0.66. **p < 0.01. (E) Real‐time qPCR analysis of *APP* gene expression in control (Ctr) and Dox‐treated (Dox) groups following bacterial challenge. Data are shown as fold change relative to controls, *n* = 6, Mean ± SD. Data were analysed by two‐way ANOVA, *F* (1,26) = 8.34, *p* = 0.0077), row factor (Blank vs. *E.coli*: *F* (1,26) = 41.65, *p* < 0.0001), and column factor (Ctr vs. Dox: *F* (1,26) = 61.11, *p* < 0.0001), with Tukey's post hoc tests. Ctr versus Dox in Blank (Ctr vs. Dox: Mean difference = 0.3282, *p* < 0.0001) and *E. coli* group (Ctr vs. Dox: Mean difference = 0.1511, *p* = 0.0089); Blank vs. *E. coli* in Ctr (Blank vs. *E. coli*: Mean difference = −0.1093, *p* = 0.0424) and Dox (Blank vs. *E. coli*: Mean difference = −0.2864, *p* < 0.0001). *p < 0.05, **p < 0.01, ****p < 0.0001 (F) Realtime PCR analysis of *APP* and *RARRES2* silencing efficiency in RPE cells, *n* = 3–6, Mean ± SD. APP: Welch's ANOVA: *F* (2, 7.69) = 13.61, *p* = 0.003. Tamhane's T2 post hoc: Vehicle vs. APP shRNA, *p* = 0.001. RARRES2: One‐Way ANOVA: *F* (2,12) = 16.41, *p* = 0.0004, *R* ^2^ = 0.73. Tukey's post hoc: Vehicle versus RARRES2 shRNA, *p* = 0.0004. ***p < 0.001, ****p < 0.0001. (G) Representative images (left) and quantitative analysis (right) of extracellular bacterial colonies in empty vector (Control, (Ctr) and APP‐ or RARRES2‐shRNA of ARPE19 cells, *n* = 4, Mean ± SD, One‐way ANOVA: *F* (3, 11) = 4.045, *p* = 0.0365, *R* ^2^ = 0.5246. Tukey's post hoc: Vehicle versus APP shRNA, *p* = 0.0337. Vehicle versus RARRES2 shRNA, *p* = 0.7940. *p < 0.05, ns: No significant difference.

To understand if altered AMP/AMP precursor expression in senescent RPE cells affects bactericidal activity, healthy and senescent RPE cells were incubated with *E. coli* for 6 h, the residual bacteria in RPE cells were detected using semi‐quantitative PCR, and the bacteria in the supernatant were examined using the spread LB agar plates method (Figure 4A). The PCR results from two *E. coli‐*specific primers showed significantly more bacterial DNA in Dox‐ or H_2_O_2_‐induced senescent RPE cells (Figure 4B,C), suggesting reduced intracellular bactericidal activity. The LB agar plate culture yielded a higher bacterial load in the supernatant of senescent RPE cells, indicating impaired bacterial clearance from the culture medium by senescent RPE cells (Figure 4D).

We further found that *E. coli* treatment significantly upregulated *APP* gene expression in normal and senescent RPE cells (Figure 4E). The treatment did not affect *RARRES2* gene expression (data not shown).

To further understand whether *APP* and *RARRES2* contribute to the anti‐bacterial activity of RPE cells, we used shRNA to knock down these genes. The expression levels of *APP* and *RARRES2* were reduced by 50.5% and 58.5%, respectively, after relevant shRNA treatment compared with the vector controls (Figure 4F). The bactericidal assay showed that the supernatant of APP shRNA‐treated RPE cells exhibited significantly higher bacterial retention (Figure 4G). No significant difference in bacterial retention was observed between RARRES2 shRNA‐treated cells and the empty vector controls (Figure 4G).

Taken together, our results suggest the anti‐bacterial activity of RPE declines when the cells become senescent, and the expression of APP is partially responsible for the anti‐bacterial function.

### 3.5 The Expression of APP Secretases and Amyloid β in Senescent RPE Cells

**FIGURE 5:**
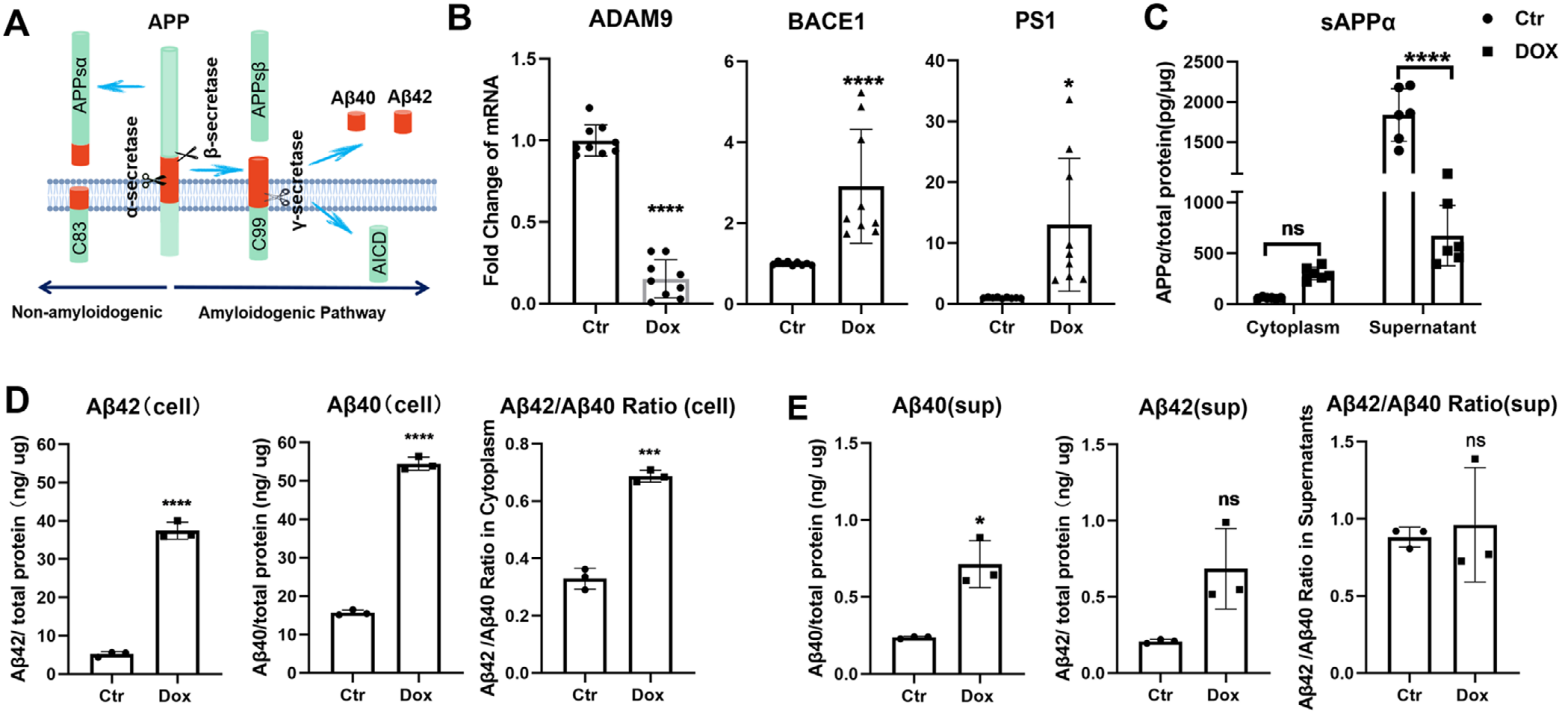
The effects of cellular senescence on APP secretase and APP cleavage products in RPE cells. (A) Schematic graph showing the amyloid precursor protein (APP) processing pathways. The non‐amyloidogenic pathway involves cleavage by α‐secretase, leading to the formation of C83 and the release of APPsα. The amyloidogenic pathway involves β‐secretase cleavage, resulting in the formation of C99, which is further processed by γ‐secretase into Aβ40 and Aβ42 peptides. (B) The mRNA expression of APP secretase *ADAM9* (one of α‐secretase), *BACE1* (β‐secretase), and *PS1* (a component of γ‐secretase) in control (Ctr) and doxorubicin (Dox)‐induced senescent ARPE19 cells using real‐time qPCR, *n* = 9, Mean ± SD. ADAM9: *F* (8, 8) = 1.516, *p* = 0.5697, unpaired *t*‐test, *t* (16) = 16.90, *p* < 0.0001. BACE1: Mann–Whitney *U* test: *U* = 0, *p* < 0.0001. PS1: *F* (8, 8) = 9536, *p* < 0.0001, Welch's corrected *t*‐test, *t* (8.002) = 3.300, *p* = 0.0109. *p < 0.05, ****p < 0.0001, Unpaired Student *t*‐test. (C) Quantification of sAPPα protein levels in ARPE19 cell lysates and the supernatant in control and Dox‐induced senescent RPE cells using ELISA assay (data were normalised to total protein), *n* = 6, Mean ± SD. Data were analysed with Two‐way ANOVA (*F* (1,20) = 59.25, *p* < 0.0001), row factor (Cytoplasm vs. Supernatant: *F* (1,20) = 138.2, *p* < 0.0001), and column factor (Ctr vs. DOX: *F* (1,20) = 25.68, *p* < 0.0001). Tukey's post hoc tests confirmed significant differences between Ctr and DOX treatments in Cytoplasm (mean difference = −239.9, *p* = 0.2762) and Supernatant (mean difference = 1164, *p* < 0.0001). ****p < 0.0001, ns: Not significant. (D, E) Quantification of Aβ42 and Aβ40 protein levels in ARPE19 cell lysates (D) and the supernatant (E) in control and Dox‐induced senescent RPE cells using ELISA assay (data were normalised to total protein), *n* = 3, Mean ± SD, Aβ42(cell): *F* (2, 2) = 10.66, *p* = 0.1715, unpaired *t*‐test, *t* (4) = 24.03, *p* < 0.0001. Aβ40(cell): *F* (2, 2) = 6.039, *p* = 0.2841, unpaired *t*‐test, *t* (4) = 35.84, *p* < 0.0001. Aβ42/Aβ40 Ratio(cell): *F* (2, 2) = 30,117, *p* = 0.4858, unpaired *t*‐test, *t* (4) = 14.81, *p* = 0.0001. Aβ42(sup): *F* (2, 2) = 420.6, *p* = 0.0047, Welch‐corrected *t*‐test, *t* (2.01) = 3.119, *p* = 0.0887. Aβ40(sup): *F* (2, 2) = 435.6, *p* = 0.0046, Welch‐corrected *t*‐test, *t* (2.01) = 5.404, *p* = 0.0323. Aβ42/Aβ40 Ratio(sup): *F* (2, 2) = 32.51, *p* = 0.0597, unpaired *t*‐test, *t* (4) = 0.3664, *p* = 0.7326. *p* < 0.0001. *p < 0.05, **p < 0.01, ns: Not significant.

Amyloid β (Αβ) is known to play an important role in age‐related neurodegenerative diseases, including age‐related macular degeneration (Prasad et al. 2017). APP, the precursor of Aβ, can be cleaved by three proteases, α‐, β‐, and γ‐secretases (Figure 5A). The α‐secretase cleaves the APP towards the non‐amyloidogenic pathway, whereas the β‐ and γ‐secretases drive the APP towards the amyloidogenic pathway (Figure 5A). We found that the expression of α‐secretase (*ADAM9*) was significantly reduced, whereas the expression of β‐secretase (*BACE1*) and γ‐secretase (*PS1*) was significantly increased in senescent RPE cells (Figure 5B). The soluble sAPPα (sAPPα) generated by α‐secretase was significantly reduced in the supernatant of senescent cells (Figure 5C). The β‐ and γ‐secretases enzymatically break APP into soluble Aβ monomers containing 39–43 amino acids. Among them, Aβ40 and Aβ42 are the two most common isoforms (Haass and Selkoe 2007). The high ratio of Aβ42/40 is critically involved in late tau pathology and other neurodegenerative diseases (Kwak et al. 2020). We found that the Aβ40 and Aβ42 proteins were significantly increased in both the cell lysates (Figure 5D) and supernatants (Figure 5E) of senescent RPE cells. The Aβ42/Aβ40 ratio was also significantly higher in senescent cells (Figure 5D) but not in the supernatants (Figure 5E). Our results suggest that senescent RPE cells are programmed to generate Aβ due to the downregulation of α‐secretase and upregulation of β/γ‐secretases.

### 3.6 Accumulation of Aβ42 in RPE Cells From Aged Donors

**FIGURE 6:**
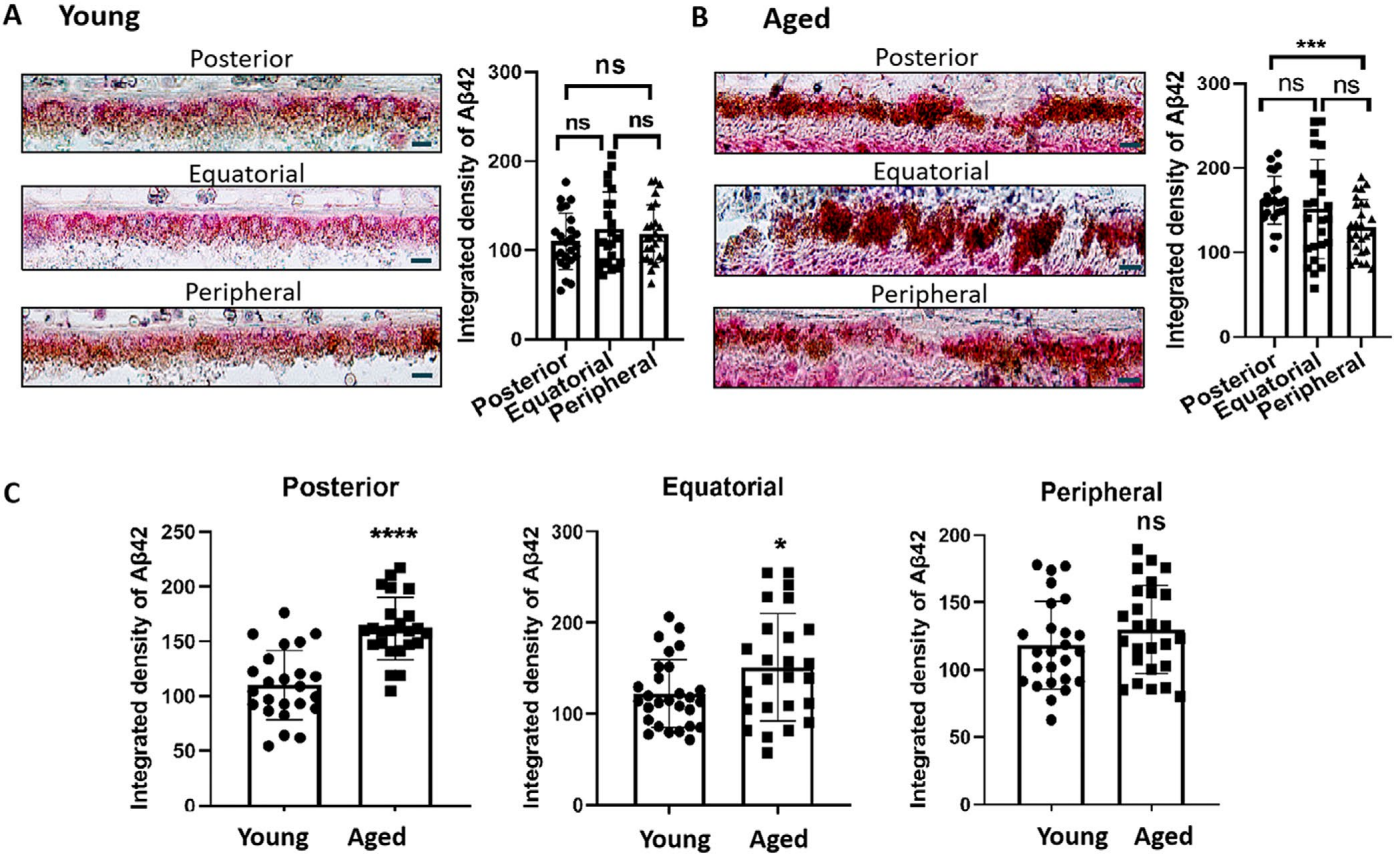
The Aβ42 expression in different regions of retinal pigment epithelium (RPE) cells in young and old human donor eyes. (A, B) Immunohistochemical analysis of Aβ42 in the posterior, equatorial, and peripheral regions of the RPE cells from young (20–50 years old) (A) and Aged (60–90 years old) (B) donors. Young: 6 sections from 4 eyes, Mean ± SD, One‐Way ANOVA: *F* (2, 68) = 0.8637, *p* = 0.4262. Tukey's post hoc: Posterior versus Equatorial, *p* = 0.4021; Posterior versus Peripheral, *p* = 0.6868; Equatorial versus Peripheral, *p* = 0.872. Aged: 5 sections from 5 eyes, Mean ± SD, Welch's ANOVA: *F* (2.000, 45.60) = 6.624, *p* = 0.0030, Games–Howell post hoc: Posterior versus Equatorial, *p* = 0.7154; Posterior versus Peripheral, *p* = 0.0018; Equatorial versus Peripheral, *p* = 0.2618. Scale bar = 20 μm. (C) Quantification of Aβ42 expression in the posterior, equatorial, and peripheral parts of the RPE in young and old donor eyes. Young: 6 sections from 4 eyes; aged: 5 sections from 5 eyes. Mean ± SD, Posterior: *F* (23, 23) = 1.239, *p* = 0.6110, unpaired *t*‐test, *t* (46) = 5.936, *p* < 0.0001. Equatorial: *F* (24, 27) = 2.517, *p* = 0.0218, Welch‐corrected *t*‐test, *t* (39.61) = 2.115, *p* = 0.0407. Peripheral: *F* (25, 24) = 1.020, *p* = 0.9637, unpaired *t*‐test, *t* (49) = 1.276, *p* = 0.2081. *p < 0.05, ***p < 0.001, ****p < 0.0001, ns = not significant.

We further investigated the expression of Aβ42 in RPE cells in human eyes from young (20–50 years old) and aged (60–90 years old) donors using immunohistochemistry. Aβ42 was detected in different regions of RPE cells in young and aged donor eyes (Figure 6A,B). The expression levels did not differ between the peripheral, equatorial, and posterior regions of the RPE in young donor eyes (Figure 6A). In aged donor eyes, the Aβ42 expression in the posterior region was significantly higher than that in the peripheral region (Figure 6B). The RPE cells from the posterior and equatorial regions of the aged donor eyes had significantly higher levels of Aβ42 compared to the cells of the same regions from young donor eyes (Figure 6C). The regional difference in Aβ42 accumulation in aged donor eyes may reflect different levels of oxidative stress and RPE senescence in different parts of the retina.

## 4 Discussion

In this study, we show that RPE cells express a variety of AMPs under physiological conditions. The AMP precursors APP and chemerin, in particular, are expressed at high levels, and their expression is reduced during ageing and in senescent RPE cells. This reduction is accompanied by increased intracellular bacterial retention in senescent RPE cells. Importantly, we show that senescence upregulates the expression of β‐secretase (*BACE1*) and γ‐secretase (*PS1*) and induces intracellular amyloid‐β deposition in RPE cells. Our results suggest that senescence compromises the antimicrobial function of the oBRB and promotes amyloid‐β deposition in RPE cells. RPE cells segregate the neuronal retina from the choroidal blood circulation and are critical for retinal homeostasis. They express and produce a variety of immune regulatory molecules such as Fas ligand (FasL), TGFβ, HMGB‐1 (Kazama et al. 2008), CTLA2, CTLA4, neuropeptides (Mochizuki et al. 2013), and complement inhibitors such as CFH (Chen et al. 2019) to prevent overt inflammation. We show that RPE cells constitutively produce various AMPs/AMP precursors such as APP and chemerin, and their expression is altered in inflammatory/infectious conditions (Figure S1). We previously showed that RPE cells express high levels of lysozyme, and knockout of lysozyme (*Lyz*) impaired the bactericidal function of RPE cells (Liu et al. 2021). In this study, we further found that the reduced expression of APP is accompanied by impaired bactericidal function in RPE cells. Our results suggest that the RPE‐derived AMPs are functional and may protect the retina from blood‐borne pathogens.

Interestingly, the expression of different AMP/AMP precursor genes in RPE cells is regulated by different TLR agonists (Figure S1). For example, the TLR3 agonist Poly(I:C) upregulated *CAMP* and *HAMP* but downregulated *RARRES2* expression in RPE cells. TLR3 activation is linked to anti‐viral/anti‐microbial responses, partially through enhancing the AMP, Cathelicidin (LL37 encoded by *CAMP* gene) in epithelial cells (Takiguchi et al. 2014). Pam3CSK4, a TLR2/TLR1 agonist, selectively increased *RARRES2* (Chemerin) and suppressed *FAM3A* and *GNLY* gene expressions. *RARRES2* encodes a 16 kDa inactive form of Chemerin. The C‐terminal fragment (e.g., chemerin 15, 2.2 kDa) is released via proteolysis and has strong antibacterial and antifungal activity (Banas et al. 2013). Our results suggest that when TLR2/TLR1 is activated at the oBRB, *RARRES2* is upregulated as part of the mechanism to eliminate pathogens.

When RPE cells undergo senescence, the *APP* and *RARRES2* (Chemerin) expressions are downregulated, whereas the expression of other AMPs (*FAM3A, CAMP, HAMP*) is upregulated. This is in line with a previous study, whereby the authors reported the downregulation of APP and its processing in senescent human fibroblasts due to age‐associated cellular cholesterol (Kern et al. 2006). The upregulation of other AMPs may be related to the proinflammatory microenvironment of senescent cells (i.e., senescence‐associated secretory phenotype, SASP). For instance, *CAMP* (cathelicidin) is known to be induced by DNA damage and NF‐κB activation (Niyonsaba et al. 2005).

Aβ can act as a natural antibiotic in the brain against common and clinically relevant microorganisms, including *Candida albicans* , *E. coli* , *S. aureus* , and *S. pneumoniae* (Soscia et al. 2010), and viruses (e.g., HSV and HIV) (Fulop et al. 2019). We found that knocking down the *APP* mRNA by 50% reduced the bactericidal activity of RPE cells (Figure 4). Surprisingly, although senescent RPE cells had higher intracellular Aβ production (Figure 5), their bactericidal function was reduced (Figure 4D). This may be explained by the reasons below. First, senescent cells are known to have reduced bacterial adherence and internalisation (Robledo et al. 2023). Second, the inflammatory environment of senescent cells (SASPs) may affect the intracellular growth of bacteria. Higher levels of inflammatory cytokines such as IL‐1β and IL‐6 can promote intracellular growth of bacteria (Meduri 2002). Chemerin, also known as retinoic acid receptor responder protein 2 (RARRES2), has chemotaxis and adipogenesis functions. Its proteolytic C‐terminal fragment (chemerin 15, 2.2 kDa) has been identified to have direct antimicrobial activity against a range of microorganisms, including *E. coli*, *P. aeruginosa* , and *S. aureus* (Banas et al. 2013; Sozzani et al. 2024). The decreased chemerin expression during ageing and in senescence would affect the chemotaxis and antimicrobial activities of RPE cells. Lysozyme is particularly effective against Gram‐positive bacteria due to its ability to break down their peptidoglycan cell walls. In addition to the antimicrobial activities, these peptides can also act as mediators of inflammation with effects on tissue‐resident cells and infiltrating cells, influencing cell proliferation, wound healing, cytokine/chemokine production, and chemotaxis. The reduced expression of APP and chemerin in senescent RPE cells suggests an impaired defence function, which may contribute to chronic inflammation in the ageing retina (Chen et al. 2019).

APP‐derived sAPPα can regulate synapse formation and repair, neural plasticity, iron export, and mitochondrial function (Lopez Sanchez et al. 2019), and is the source of Aβ (Ristori et al. 2020). Senescence not only reduced the expression of APP and the production of sAPPα but also promoted the generation and intracellular accumulation of Aβ, particularly the pathogenic fragment Aβ42, evidenced by increased Aβ42/Aβ40 ratio in the cell lysates (Figure 5). Generation of sAPPα through α‐secretase of the non‐amyloidogenic pathway reduces the β‐secretase activity and Aβ generation (Ristori et al. 2020). The declines in APP expression (Figure 3C) and α‐secretase (Figure 5B) in senescent cells may shift APP processing towards the amyloidogenic pathway, driving Aβ deposition. It should be noted that this study only measured the mRNA of the β‐secretase (BACE1) and γ‐secretase key component (PS1). We did not measure the proteins, and more importantly, the enzyme activities of these secretases. In addition to these enzymes, the Aβ generation is also regulated by the lipid membrane microenvironment (Urano et al. 2005), post‐translational modifications of BACE1, and other γ‐secretase subunits, including nicastrin, APH‐1, and PEN‐2 (Takasugi et al. 2003). Further investigation on how the senescent microenvironment affects the β/γ‐secretases will be crucial to fully understand the mechanism of Aβ accumulation in RPE cells under ageing conditions.

Excessive Aβ accumulation has been implicated in the aetiology of Alzheimer's disease (AD) (Kwak et al. 2020) and age‐related macular degeneration (AMD) (Yoshida et al. 2005). Patients with AD often have signs of retinal degeneration, including the loss of neurons and retinal thinning (Guo et al. 2010). Aβ has been detected in sub‐RPE deposits in older donors (Löffler et al. 1995) and drusen in AMD patients (Dentchev et al. 2003). Previous studies have shown that Aβ42 can initiate inflammation and induce RPE cell death and retinal degeneration (Kurji et al. 2010). Neutralising Aβ with a monoclonal antibody attenuated retinal pathologies in a mouse model of AMD (Ding et al. 2008), although a randomised phase 2 study did not demonstrate a therapeutic effect in AMD patients with geographic atrophy (dry AMD) (Rosenfeld et al. 2018), highlighting the complexity of Aβ in AMD pathologies. The source of Aβ42 in the ageing retina and AMD eyes remained elusive. Using both RPE cultures and donated human eyes, we demonstrated that RPE cells can produce Aβ, particularly under ageing and in senescent conditions, due to the shift to β−/γ‐secretases from α‐secretase (Figure 5). Why senescence reduces the expression of α‐secretase and increases the expression of β‐ and γ‐secretases warrants further investigation.

## 5 Conclusions

We demonstrated the antimicrobial activity of RPE cells as a novel function of the oBRB to protect the neuroretina from blood‐borne pathogens. APP and chemerin represent key AMP precursors that can generate AMPs for antimicrobial function. The antimicrobial activity declines with age and is impaired in senescent RPE cells, putting the ageing retina at risk of low‐grade chronic inflammation and neurodegeneration (Chen et al. 2019; Xu et al. 2009). Senescence also induces intracellular Aβ deposition in RPE cells, which may play an important role in the development and progression of various types of age‐related retinal degeneration.

## Author Contributions

H.X.: conceptualisation and study design; J.L.: experimentation and data acquisition; C.Y. and M.C.: bioinformatics and data analysis; J.Q. and X.Y.: assistance in experiments and data acquisition; X.C.: image analysis and quantification. All authors have read and approved the final version and are responsible for the content of the release. All authors participated sufficiently in the work and agreed to take responsibility for all aspects of the work.

## Disclosure

Part of the work has been presented at the 2024 ARVO annual meeting in May 2024.

## Conflicts of Interest

The authors declare no conflicts of interest.

## Supporting information


Figure S1.

## Data Availability

The single‐cell sequencing data analysed in this study were derived from publicly available datasets accessed via the following resources: Spectacle: https://singlecell‐eye.org/app/spectacle/ (Dataset: GSE210543); Single Cell Portal: https://singlecell.broadinstitute.org/ (Dataset: GSE135133). All other data generated or analysed during this study are included in this published article and its Supporting Information files.
