# Supplementary material for: Senescence Alters Antimicrobial Peptide Expression and Induces Amyloid‐β Production in Retinal Pigment Epithelial Cells
Source: Aging Cell. 2025 Jul 13;24(9):e70161. doi: 10.1111/acel.70161 (PMC12419862; doi:10.1111/acel.70161)
Supplement: Supplementary file 1 — Figure S1. [file ACEL-24-e70161-s001.docx]

**Supplementary Figure S1**





**Supplementary Figure S1**. The effect of inflammatory conditions on AMP mRNA expression in ARPE19 cells. ARPE19 cells were treated with LPS（100 ng/mL), Pam3CSK (2 ng/mL), PGN-SA ( 10 μg/mL), naked Poly(I:C) (5 μg/mL), naked Poly(dA:dT) (5 μg/mL), Poly(I:C) transfected with lipofectamine 2000 (Lipo2000), and Poly(dA:dT) (5 μg/mL) transfected with Lipo2000 for 24 hours. The expression of AMP mRNA was examined by real-time qPCR. N = 3, Mean ± SD, *P < 0.05, **P < 0.01, ***P < 0.001, ****P < 0.0001 compared to control group (Ctr); ns (not significant). One-way ANOVA with Dunnett’s multiple comparison tests.
